# P-2007. Prognosis of COVID-19 Patients with Preexisting Dementia: Findings from a Comprehensive Multicenter Propensity-Matched Cohort Study in Brazil

**DOI:** 10.1093/ofid/ofae631.2164

**Published:** 2025-01-29

**Authors:** Victor Schulthais Chagas, Lucas Emanuel F Ramos, Magda Carvalho Pires, Patryk Marques da Silva Rosa, Virginia Mara Reis Gomes, Joice Coutinho de Alvarenga, Karina Cardoso Meira, Márlon Juliano Romero Aliberti, Maria Aparecida Camargos Bicalho, Milena S Marcolino

**Affiliations:** Universidade Federal de VIçosa, Viçosa, Minas Gerais, Brazil; Universidade Federal de Minas Gerais, Belo Horizonte, Minas Gerais, Brazil; Federal University of Minas Gerais, Belo Horizonte, Minas Gerais, Brazil; Hospital das Clínicas - Universidade Federal de Minas Gerais, Belo Horizonte, Minas Gerais, Brazil; Universidade Federal de Minas Gerais, Belo Horizonte, Minas Gerais, Brazil; Hospital João XXIII, Belo Horizonte, Minas Gerais, Brazil; Universidade Federal do Rio Grande do Norte, Natal, Rio Grande do Norte, Brazil; Hospital das Clinicas HCFMUSP, Medical School, Universidade de Sao Paulo; Research Institute, Hospital Sirio-Libanes, São Paulo, Sao Paulo, Brazil; Hospital João XXIII, Belo Horizonte, Minas Gerais, Brazil; Medical School, Universidade Federal de Minas Gerais, Belo Horizonte, Minas Gerais, Brazil

## Abstract

**Background:**

Despite the increasing knowledge about Covid-19 manifestations, there is still a lack of studies regarding the impact of the pandemic in patients with dementia. This study aims to evaluate clinical characteristics and outcomes of patients with dementia hospitalized due to Covid-19.

**Methods:**

This retrospective observational study is part of a multicentric cohort that analyzed data from 38 hospitals from 19 Brazilian cities. Patients were included if they were ≥ 60 years old and hospitalized due to laboratory-confirmed Covid-19 between March 2020 and March 2022. Demographics data, clinical characteristics at admission, laboratory findings and outcomes were collected. Patients were matched in a propensity score analysis according to the presence or absence of dementia and stratified by age, sex, number of comorbidities, hospital, and year. A logistic regression model to estimate the propensity score was employed.

**Results:**

Overall, 1,556 patients were included (median age 82 [interquartile range [IQR] 76-87 years], 58.7% women). A total of 405 had dementia diagnosed in medical records or by statement. Dementia group presented a higher prevalence of dyspnoea, cough, myalgia, headache, anosmia and ageusia compared to those without dementia. At hospital admission, dementia group had a higher prevalence of delirium (38.5% vs. 22.5%; p< 0.001), fever (49.4% vs. 41.7%; p=0.009), sensory impairment (57.3% vs. 25.6%; p< 0.001) and lower frequency of invasive mechanical ventilation support (23% vs. 37.8%; p< 0.0001). Palliative care was more frequent in dementia patients when compared to controls (39.8% vs. 19.7%; p< 0.001). There were no clinically relevant differences in laboratory results. Regarding outcomes, those with dementia had shorter length of stay in hospital (7.0 vs. 9.0 days; p=0.026), lower frequency of sepsis (17.0% vs. 24.0%; p=0.005) and kidney replacement therapy (6.4% vs. 9.8; p=0.002). There was no difference observed in hospital mortality between groups.

**Conclusion:**

Clinical characteristics of Covid-19 differ between older patients with and without dementia. Dementia alone cannot explain the higher short-term mortality due to Covid-19. Other risk factors such as frailty and acute morbidity should be considered.

**Disclosures:**

All Authors: No reported disclosures

